# Tin Complexes of 4-(Benzylideneamino)benzenesulfonamide: Synthesis, Structure Elucidation and Their Efficiency as PVC Photostabilizers

**DOI:** 10.3390/polym13152434

**Published:** 2021-07-23

**Authors:** Hassan Ghani, Emad Yousif, Dina S. Ahmed, Benson M. Kariuki, Gamal A. El-Hiti

**Affiliations:** 1 Department of Chemistry, College of Science, Al-Nahrain University, Baghdad 64021, Iraq; st.hassan.ghani@nahrainuniv.edu.iq (H.G.); emad.yousif@nahrainuniv.edu.iq (E.Y.); 2 Department of Medical Instrumentation Engineering, Al-Mansour University College, Baghdad 64021, Iraq; dina.saadi@muc.edu.iq; 3 School of Chemistry, Cardiff University, Main Building, Park Place, Cardiff CF10 3AT, UK; kariukib@cardiff.ac.uk; 4 Department of Optometry, College of Applied Medical Sciences, King Saud University, P.O. Box 10219, Riyadh 11433, Saudi Arabia

**Keywords:** poly(vinyl chloride), photostabilization, 4-(benzylideneamino)benzenesulfonamide, weight loss, organotin complexes, surface morphology

## Abstract

Poly(vinyl chloride) (PVC) suffers from photo-oxidation and photodegradation when exposed to harsh conditions. Application of PVC thus relies on the development of ever more efficient photostabilizers. The current research reports the synthesis of new complexes of tin and their assessment as poly(vinyl chloride) photostabilizers. The three new complexes were obtained in high yields from reaction of 4-(benzylideneamino)benzenesulfonamide and tin chlorides. Their structures were elucidated using different tools. The complexes were mixed with poly(vinyl chloride) at a very low concentration and thin films were made from the blends. The effectiveness of the tin complexes as photostabilizers has been established using a variety of methods. The new tin complexes led to a decrease in weight loss, formation of small residues, molecular weight depression, and surface alteration of poly(vinyl chloride) after irradiation. The additives act by absorption of ultraviolet light, removal the active chlorine produced through a dehydrochlorination process, decomposition of peroxides, and coordination with the polymeric chains. The triphenyltin complex showed the greatest stabilizing effect against PVC photodegradation as a result of its high aromaticity.

## 1. Introduction

Plastic is a polymeric material that is widely used as an alternative for wood, glass, and metals in various applications [1,2]. Plastics are cheap to produce and have superior performance and unique properties compared with other materials [3]. Everyday applications include use in vehicles (e.g., airbags, child safety seats, and bicycle helmets), electronic equipment (e.g., computers, cell phones, and televisions), building construction (e.g., insulation, flooring, walls, and roofs), toys, and packaging. Polymers can occur in nature (e.g., rubber) or can be produced synthetically as in the case of poly(vinyl chloride) (PVC). They can be soft and flexible owing to low crystallinity or rigid with a high level of crystallinity [4].

PVC is a thermoplastic that is suitable for many applications thanks to its excellent performance and unique properties. Among synthetic polymers, it is only superseded by polyethylene and polypropylene in terms of production and consumption scales [5]. PVC is non-combustible as 57% of its weight is chlorine, and has many qualities that make it attractive for various outdoor applications [6]. However, sunlight and harsh conditions have a detrimental effect on PVC, leading to its photooxidation and photodegradation. The weathering of PVC takes place at temperatures above 100 °C [7,8]. PVC contains only saturated bonds (C–Cl, C–H, and C–C), and thus does not absorb UV light with a wavelength higher than 220 nm [9]. However, the defects (e.g., polyene residues) within the PVC polymeric chains can initiate the photodegradation process [10]. Photodegradation of PVC due to ultraviolet (UV) irradiation is complex and various mechanisms have been suggested [11,12]. It causes production of chlorine radicals (Cl**·**) and hydrogen chloride (HCl). Dehydrochlorination leads to production of unsaturated residues (e.g., (–CH=CH–)*_n_*) in polymeric materials. In addition, it initiates oxidation and decomposition of PVC and leads to discoloration (dark brownish color); cracks; and a reduction in durability, weight, and strength [13,14,15]. Photooxidation of PVC leads to cross-linking, chain scission, and formation of peroxides and carbonyl fragments. Thus, in order to preserve the long-term integrity of PVC, a number of additives are used to enhance its photostability, particularly for outdoor applications [16,17].

PVC additives include fire and flame retardants, heat and energy quenchers, radical and peroxide scavengers, UV absorbers, and antioxidants [18,19]. The additives need to be safe, be benign to the environment and health, have no effect on the color or properties of PVC, be easy and cheap to produce, be used at a low concentration, and be able to blend well with the polymer. Common industrial PVC stabilizers include polychlorinated biphenyls (flame retardants), phthalic acids (plasticizers), phosphites (stabilizers), and metals containing additives [20,21,22,23]. However, many of these additives are toxic to humans, not safe to the environment, not suitable for PVC used in medicinal applications such as blood bags, or require the use of co-stabilizers. There is thus a need for the design and synthesis of safer and efficient PVC stabilizers.

Several organic- and inorganic-based stabilizing materials have been used to enhance the stability of PVC. The most common PVC stabilizers include Schiff bases [24,25,26,27,28], polybenzimidazoles [29], polyphosphates [30], pigments [31], zinc oxide [32], and titanium dioxide [33,34]. Tin complexes containing organic moieties have high stability and are used in many medicinal and industrial applications [35,36,37,38]. The efficiency of tin complexes is highly dependent on the organic moieties and substituents attached to the tin (Sn) atom [39]. Several tin complexes containing different aromatic moieties have been used to stabilize PVC against UV irradiation [40,41,42,43]. For example, tin complexes containing naphthalene sulfonic acid have been proven to be efficient in reducing the roughness factor (*R*q) of PVC blends when irradiated with UV light for durations of up to 300 h [43].

The aim of this work was to explore the potential of tin complexes containing the 4-(benzylideneamino)benzenesulfonamide to act as efficient PVC stabilizers. 4-(Benzylideneamino)benzenesulfonamide has never been used in the production of PVC additives. Such a ligand contains two aromatic rings, nitrogen, oxygen, and sulfur, and can act as a primary and secondary stabilizer. In addition, the Sn atom acts as a HCl scavenger. The synthesis of new tin complexes containing 4-(benzylideneamino)benzenesulfonamide is reported and their ability to reduce the photooxidation and photodegradation of PVC is assessed.

## 2. Materials and Methods

### 2.1. Instruments and Chemicals

PVC (degree of polymerization = 800 and K-value = 67) was purchased from Petkim Petrokimya (Istanbul, Turkey). 4-(Benzylideneamino)benzenesulfonamide (ligand; 98%), triphenyltin chloride (Ph_3_SnCl; 95%), tri-*n*-butyltin chloride (Bu_3_SnCl; 96%), and dimethyltin dichloride (Me_2_SnCl_2_; 97%) were purchased from Merck (Gillingham, UK). The melting points were recorded on a Gallenkamp apparatus (Calgary, AB, Canada). The elemental content of the complexes was determined using a Vario EL III elemental analyzer (Elementar Americas, Ronkonkoma, NY, USA). Fourier-transform infrared (FTIR) spectra (400–4000 cm^–1^) were recorded on a Shimadzu 8400 spectrophotometer (Kyoto, Japan). ^1^H (500 MHz), ^13^C (125 MHz), and ^119^Sn (149 MHz) nuclear magnetic resonance (NMR) spectra were recorded on a Bruker DRX-500 NMR spectrometer (Zürich, Switzerland) in deuterated dimethyl sulfoxide (DMSO-*d*_6_). The PVC viscosity was measured using an Ostwald U-Tube Viscometer (Ambala, India). The optical images of the complexes were captured using a Meiji Techno microscope (Tokyo, Japan). The field emission scanning electron microscope (FESEM) images were captured using a TESCAN-MIRA3 system (Kohoutovice, Czech Republic) at 10 kV. A Bruker XFlash 6 10 (Tokyo, Japan) was used to record the energy dispersive X-ray (EDX) spectra. The PVC blends were coated with gold (Au; ca. 15 nm) before the EDX was carried out. Atomic force microscopy (AFM) images were recorded on a Veeco instrument (Plainview, NY, USA). The PVC films were irradiated with a Q-Panel UV-accelerated weathering tester (Homestead, FL, USA) at room temperature.

### 2.2. Synthesis of Complexes ***1** and **2***

**Scheme 1:**
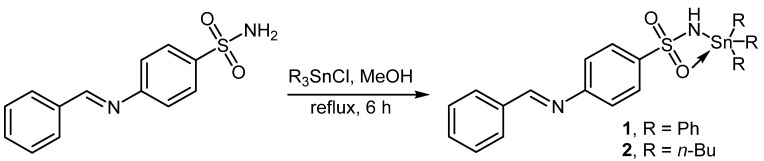
Synthesis of **1** and **2**.

A mixture of ligand (0.26 g, 1.0 mmol) and Ph_3_SnCl (0.39 g, 1.0 mmol) or Bu_3_SnCl (0.33 g, 1.0 mmol) in methanol (MeOH; 30 mL) was refluxed for six hours (Scheme 1). The solid obtained was filtered, washed with MeOH, and dried to afford **1** or **2** in good yields (Table 1). The structures of **1** and **2** were confirmed using elemental analysis, FTIR, and NMR spectral data (See Section 3.1).

### 2.3. Synthesis of Complex ***3***

**Scheme 2:**
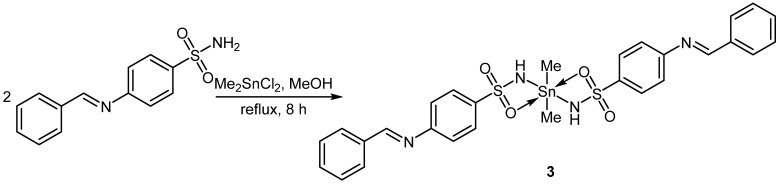
Synthesis of **3**.

A mixture of ligand (0.52 g, 2.0 mmol) and Me_2_SnCl_2_ (0.22 g, 1.0 mmol) in MeOH (30 mL) was refluxed for eight hours (Scheme 2). The solid formed was filtered, washed with MeOH, and dried to give **3** in a 76% yield (Table 1). The structure of **3** was confirmed using analytical and spectroscopic tools (Section 3.1).

### 2.4. Preparation of PVC Films

A mixture of PVC (5.0 g) and appropriate organotin complex (25 mg) in tetrahydrofuran (THF; 100 mL) was magnetically stirred for two hours at 25 °C. The mixture was poured onto a glass plate (15 holes, 4 × 4 cm^2^, and 40 µm thickness). The films obtained were left to dry for 18 h in a vacuum oven at room temperature.

### 2.5. Assessment of PVC by FTIR Spectroscopy

FTIR spectroscopy was used to investigate the efficiency of organotin complexes as PVC antidegradation additives. Irradiation of PVC blends with UV light with a λ_max_ of 313 nm for a period of 300 h leads to changes in their FTIR spectra. The intensity of absorption bands appearing at 1722 and 1604 cm^–1^, corresponding to the carbonyl group (C=O) and alkene double bonds (C=C), respectively, were monitored during irradiation [16]. The growth in the intensity of these peaks was compared with that for the C–H bond (1328 cm^–1^). The intensity of the peak due to the C–H bond is not affected by irradiation. Such a comparison provides an assessment of PVC photodegradation and production of small residues containing C=O and C=C groups [44]. The functional group index (*I_s_*) was calculated by dividing the absorbance (*A_s_*) of a group by that for the C–H peak (*A_r_*) using Equation (1).

*I_s_ = A_s_/A_r_* (1)

### 2.6. Assessment of PVC Photodegradation by Weight Loss (%)

Irradiation of PVC blends leads to the production of small molecular weight fragments. As a result, a weight loss takes place and its percentage is highly dependent on the level of photodegradation. PVC weight loss (%) due to photoirradiation was calculated from the weight of films before (*W*_1_) and after (*W*_2_) irradiation using Equation (2) [45].

*Weight loss* (%) = (*W*_1_ − *W*_2_)/*W*_1_ × 100 (2)

### 2.7. Assessment of PVC Photodegradation by Average Molecular Weight (${\bar{M}}_{V}$)

Irradiation of PVC causes a decrease in its ${\bar{M}}_{V}$ due to the formation of volatiles and small fragments. As a result, the intrinsic viscosity [*η*] of PVC drops. The depression in [*η*] is proportional to the damage occurring within PVC owing to irradiation. The ${\bar{M}}_{V}$ of irradiated PVC was calculated using the Mark–Houwink relation shown in Equation (3) [46].

(3) $$\left[\eta \right]\;=1.63\times 10^{-2}\;M_{v}^{0.77}$$

## 3. Results and Discussion

### 3.1. Synthesis of Complexes ***1**–**3***

Three new organotin complexes **1**–**3** were synthesized in 63–86% yields (Table 1) from the reaction of appropriate substituted tin chloride and 4-(benzylideneamino)benzenesulfonamide on refluxing in MeOH (Scheme 1 and Scheme 2). The purity of **1**–**3** was confirmed from the elemental composition (Table 1) and their structures were established (Table 1, Table 2, Table 3 and Table 4).

### 3.2. Characterization of Complexes ***1**–**3***

The FTIR spectra of **1**–**3** showed the presence of absorption peaks corresponding to the NH (3459–4361 cm**^–1^**), CH=N (1667–1684 cm**^–1^**), and C=C (1623–1630 cm**^–1^**) groups (Table 2). In addition, they showed the presence of SO_2_ asym and SO_2_ sym that appeared in the 1304–1307 cm**^–1^** and 1179–1188 cm**^–1^** regions, respectively. The absorption bands corresponding to Sn–O and Sn–N appeared in the 533–552 cm**^–1^** and 439–454 cm**^–1^** regions respectively, which is consistent with the literature [47,48]. The FTIR spectra of the ligand and complexes **1**–**3** are shown in Figures S1–S4.

The ^1^H NMR spectra of **1**–**3** showed the presence of aromatic, NH, and substituent (phenyl, *n*-butyl, and methyl) protons (Table 3). In addition, the ^13^C NMR spectra confirmed the presence of all carbons in complexes **1**–**3** (Table 4).

The ^119^Sn NMR spectra of **1** and **2** (Figures S5 and S6) showed a singlet at −227.4 ppm and −41.3 ppm, respectively. In contrast, the singlet signal appeared at −143.2 ppm for complex **3** (Figure S7). The ^119^Sn chemical shifts indicated that the coordination number for the tin atom was five in complexes **1** and **2** and six in complex **3** [49,50].

### 3.3. FESEM of Complexes ***1**–**3***

**Figure 1:**
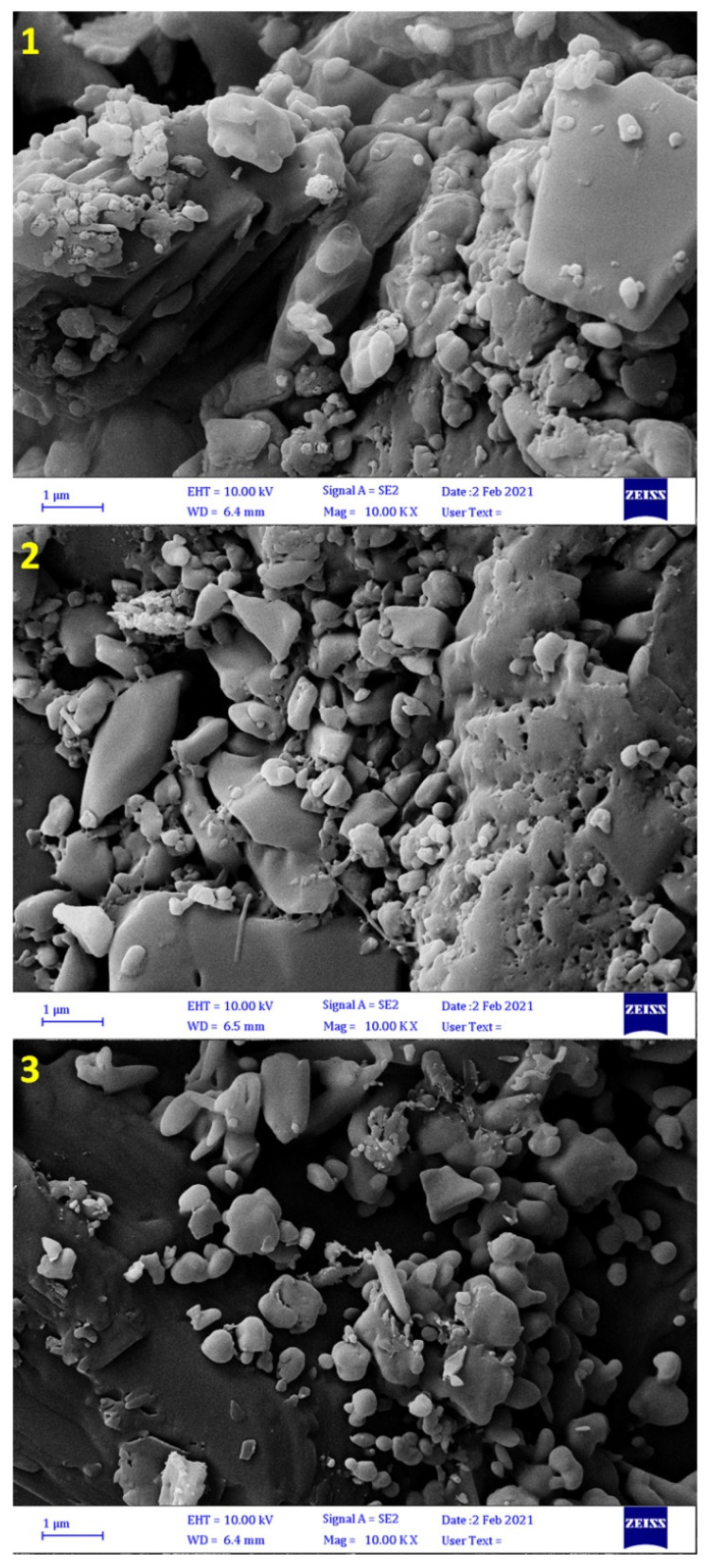
Field emission scanning electron microscope (FESEM) images of complexes **1**–**3**.

FESEM was used to assess the cross sections and variation of particle shape and size of complexes **1**–**3**. The FESEM images showed that complexes **1**–**3** have porous structures in which pores varied in shape and size. In addition, they showed the presence of some small particle agglomerates (Figure 1). The particle size of tin complexes was in the range of 480.6–664.4 nm for **1**, 167.9–864.9 nm for **2**, and 276.4–625.6 nm for **3**.

### 3.4. Assessment of PVC Photodegradation by EDX Mapping

Complexes **1**–**3** (0.5% by weight) were mixed with PVC and films (thickness of 40 µm) were produced [51]. The PVC blends were exposed to light (λ_max_ = 313 nm) for 300 h and the element content was analyzed using EDX mapping. The element composition of the blank PVC film is shown in Figure S8. The chlorine content within the PVC film dropped dramatically from 68.0% to 50.6% owing to irradiation. Photodegradation of PVC leads to bonds breaking and the release of HCl from the polymeric chains, indicating a substantial degree of dehydrochlorination [6]. After irradiation, the decrease in chlorine content (%) was low (54.2–51.1%) for the PVC blends containing complexes **1**–**3** (Figure S9). Such a result revealed that the degree of PVC photodegradation was lower in the presence of **1**–**3** than that for the pure PVC film. The highest chlorine content (54.2%) was observed with the use of complex **1**. Complex **1** contains five aromatic moieties (four phenyl and one 1,4-disubstituted benzene unit) and can absorb the UV light more efficiently.

### 3.5. Assessment of PVC Photodegradation by FTIR Spectroscopy

**Figure 2:**
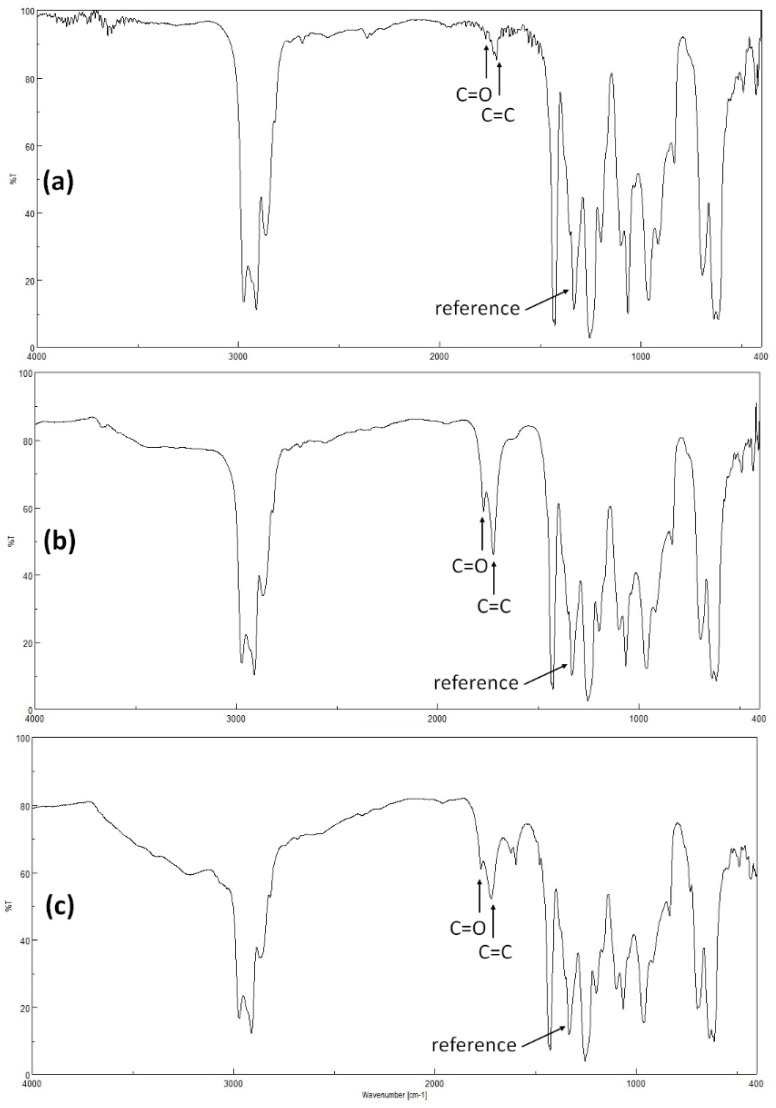
Fourier-transform infrared (FTIR) spectra of (**a**) poly(vinyl chloride) (PVC) before irradiation, (**b**) PVC after irradiation, and (**c**) PVC + **1** after irradiation.

Irradiation of PVC leads to the formation of chloride radicals responsible for the elimination of small fragments containing carbonyl (C=O) groups and alkene double (CH=CH) bonds, for example [52]. Therefore, PVC films were irradiated and the growth in the absorption peaks corresponding to C=O (1722 cm**^–1^**) and CH=CH (1604 cm**^–1^**) groups was measured and compared to that for the reference peak (C–H bond; 1328 cm**^–1^**). The FTIR spectra for the PVC (before and after irradiation) and PVC + **1** blend (after irradiation) are shown in Figure 2. The growth in the peaks for both C=O and CH=CH groups is used as a measure for the degree of PVC degradation that has taken place owing to irradiation. Figure 2 shows that the intensity of C=O and CH=CH peaks increased significantly owing to irradiation in the blank film compared with the PVC + **1** blend. Clearly, complex **1** leads to a reduction in degradation of PVC upon irradiation.

**Figure 3:**
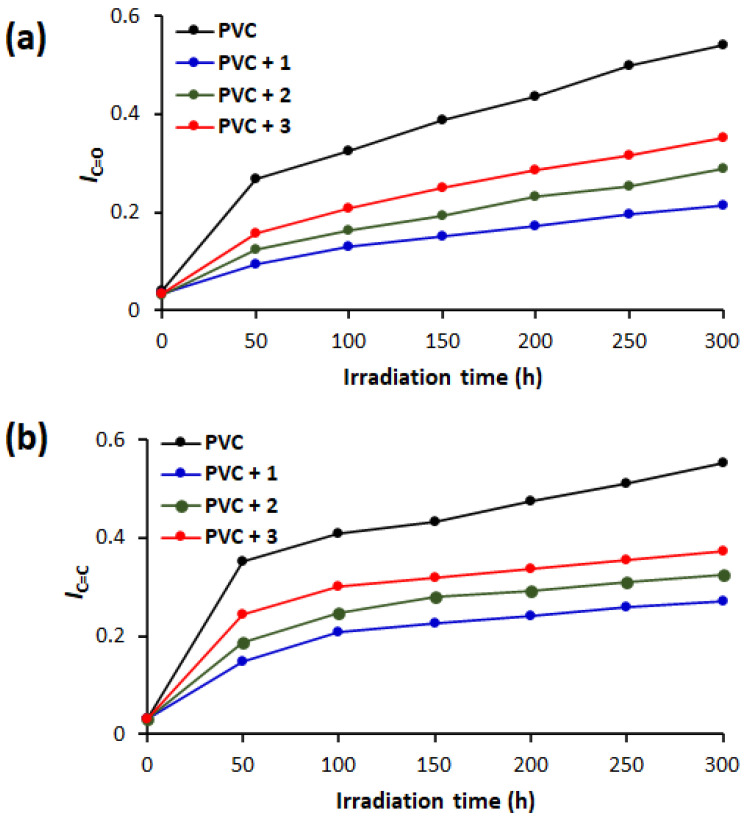
Changes in (**a**) the *I*_C=O_ and (**b**) the *I*_C=C_ of PVC films during irradiation.

The carbonyl (*I*_C=O_) and polyene (*I*_C=C_) indices were calculated using Equation (1) and plotted versus time at intervals of 50 h of irradiation. Figure 3 shows that the growth rate for the C=O and CH=CH groups was highest for the blank PVC and lowest for the blend containing **1**. For example, *I*_C=O_ and *I*_C=C_ were 0.039 and 0.030, respectively, before irradiation; 0.268 and 0.351, respectively, after 50 h of irradiation; and 0.542 and 0.553, respectively, after 300 h of irradiation. For the PVC + **1** blend, the *I*_C=O_ and *I*_C=C_ were 0.049 and 0.147, respectively, after 50 h of irradiation compared with 0.215 and 0.271, respectively, after 300 h of irradiation. Clearly, the presence of complex **1**, which has high aromaticity, substantially reduced the degradation of PVC. The effectiveness of tin complexes as PVC additives is highest for **1** (triphenyl substituted complex), followed by **2** (tributyl substituted complex), and **3** (dimethyl substituted complex) has the lowest.

### 3.6. Assessment of PVC Photodegradation by Weight Loss

**Figure 4:**
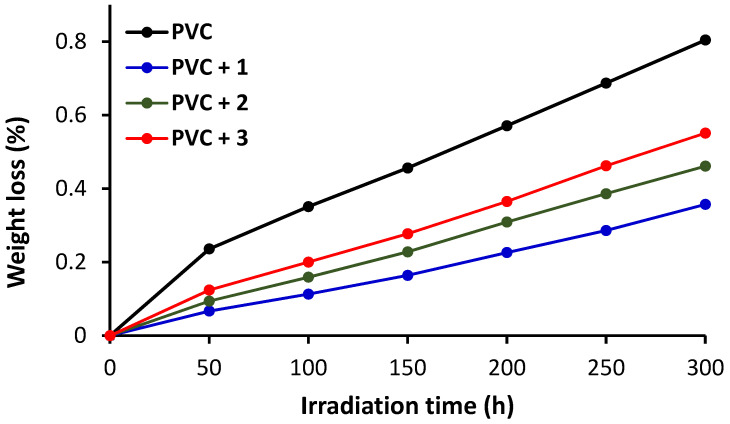
Changes in weight loss (%) of PVC films during irradiation.

Oxidation, decomposition, and degradation of PVC lead to weight loss due to elimination of free radicals and HCl, bond cleavages, cross-linking, and production of volatile small molecular weight residues [15]. The percentage weight loss from PVC is used as a tool to assess the level of photodegradation. The percentage weight loss was calculated after every 50 h of irradiation using Equation (2) and the results are shown in Figure 4. The weight loss (%) increased linearly with the time of photoirradiation in all cases. The presence of the tin complexes reduces degradation. For example, the weight loss for the pure PVC film is 0.236% after 50 h and 0.804% after 300 h of photoirradiation compared with 0.067% and 0.357%, respectively, when **1** was present. Consistent with FTIR results, complex **1** provided the most protection for PVC compared with the other two complexes (i.e., **2** and **3**).

### 3.7. Assessment of PVC Photodegradation by the ${\bar{M}}_{V}$

**Figure 5:**
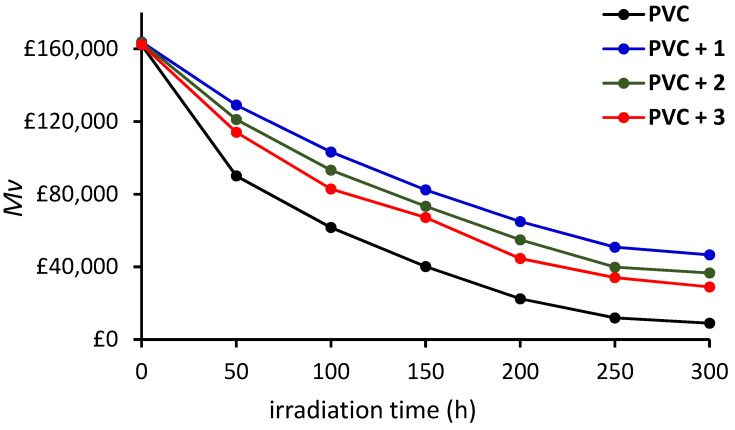
Changes in the ${\bar{M}}_{V}$ for PVC films during irradiation.

Photoirradiation of PVC causes a decrease in its ${\bar{M}}_{V}$ owing to chain scission [46,53]. A solution of irradiated PVC was made from the blends after every 50 h, in THF, and its viscosity was measured. Equation (3) was used to calculate the ${\bar{M}}_{V}$ which is presented against time (Figure 5). The decrease in the ${\bar{M}}_{V}$ was highest for the pure PVC film. The ${\bar{M}}_{V}$ for the pure PVC film decreased by 45% after the first 50 h, 86% after 200 h, and 94% at the end of the photoirradiation process. In the presence of **1**, the ${\bar{M}}_{V}$ decreased by 21% after the first 50 h, 60.3% after 200 h, and 71% after 300 h of photoirradiation. The result showed the stabilizing effect of tin complexes **1**–**3**.

### 3.8. Assessment of PVC Photodegradation by Optical Microscopy

**Figure 6:**
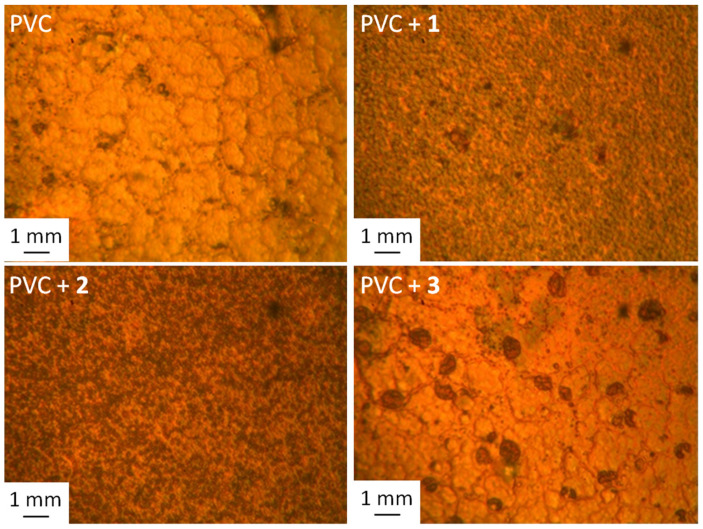
Microscope images of PVC films after irradiation at 300 h.

The surface morphology of PVC blends was investigated using a microscope to assess the damage due to photoirradiation. The optical images of the blank PVC surface before photoirradiation were smooth and regular with no cracks or discoloration. After irradiation, the surface showed cracks, white spots, darkened color, irregularity, and roughness [43,54]. The changes due to photoirradiation are mainly a result of dehydrochlorination and elimination of volatiles and breaking of bond [55]. Figure 6 shows generally rough and irregular surfaces of PVC after irradiation with the appearance of dark spots and cracks. Inspection showed that the cracks were longer and deeper in the case of blank PVC film, indicating that the use of tin complexes **1**–**3** led to some stabilization of the polymeric films against photoirradiation.

### 3.9. Assessment of PVC Photodegradation by the FESEM

**Figure 7:**
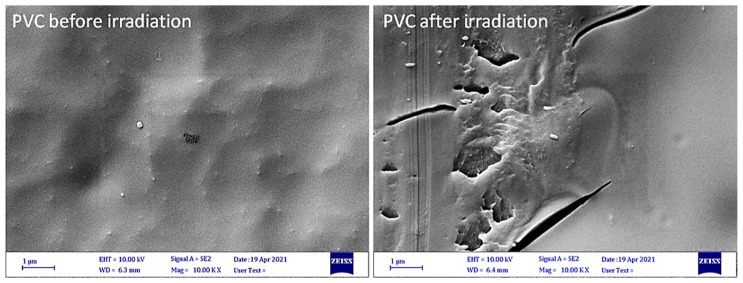
FESEM images of the blank PVC film before and after irradiation.

**Figure 8:**
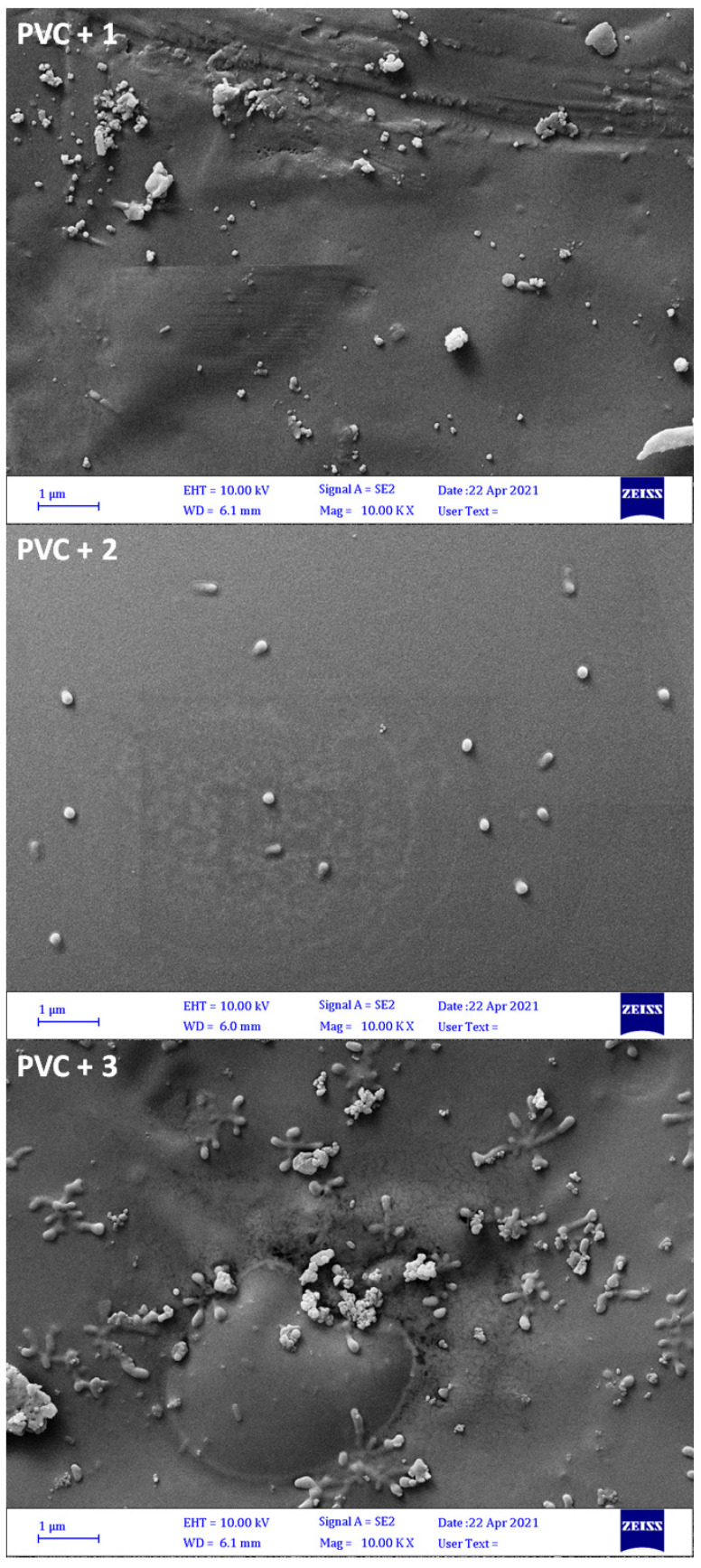
FESEM images of PVC films after irradiation.

The surface of the irradiated and non-irradiated PVC films was inspected using FESEM, which provides high resolution images. Figure 7 clearly shows that the surface of the pure PVC film was badly damaged after irradiation. The surface was irregular, not homogeneous, lumpy, and contained cracks of different shapes and sizes after irradiation. For the pure PVC film, the particles size was 94.9–190.5 nm before irradiation compared with 101.7–116.1 nm after irradiation. Figure 8 shows that the surface of films containing tin complexes **1**–**3** after irradiation was more regular, smoother, and less lumpy with a high degree of homogeneity. Clearly, additives **1** and **2** have most efficiently stabilized PVC followed by **3**. The additives substantially reduced PVC photodegradation. After irradiation (300 h), the particles size was 76.4–144.7 nm for PVC + **1**, 189.2–278.3 nm for PVC + **2**, and 66.6–146.9 nm for PVC + **3**.

### 3.10. Assessment of PVC Photodegradation by the AFM

**Figure 9:**
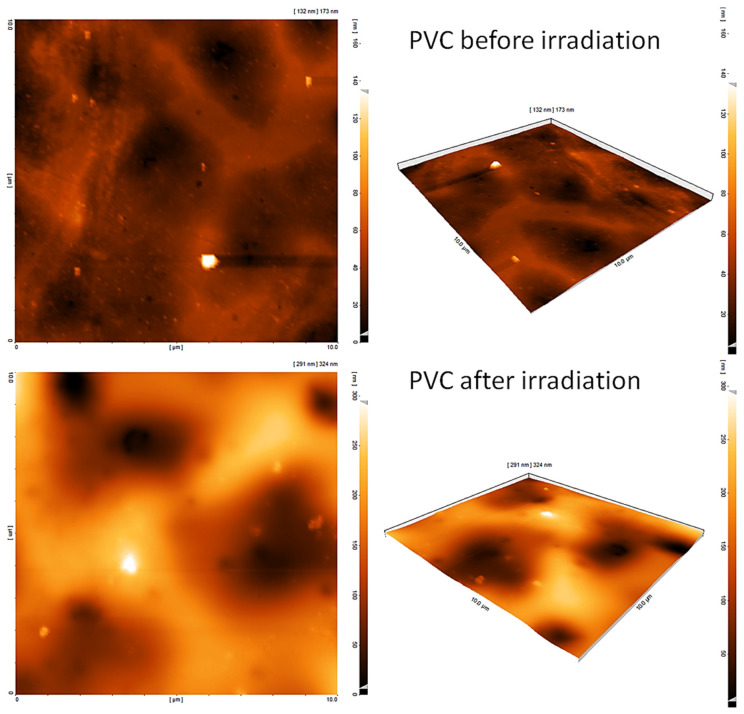
Atomic force microscopy (AFM) images of the blank PVC before and after irradiation.

**Figure 10:**
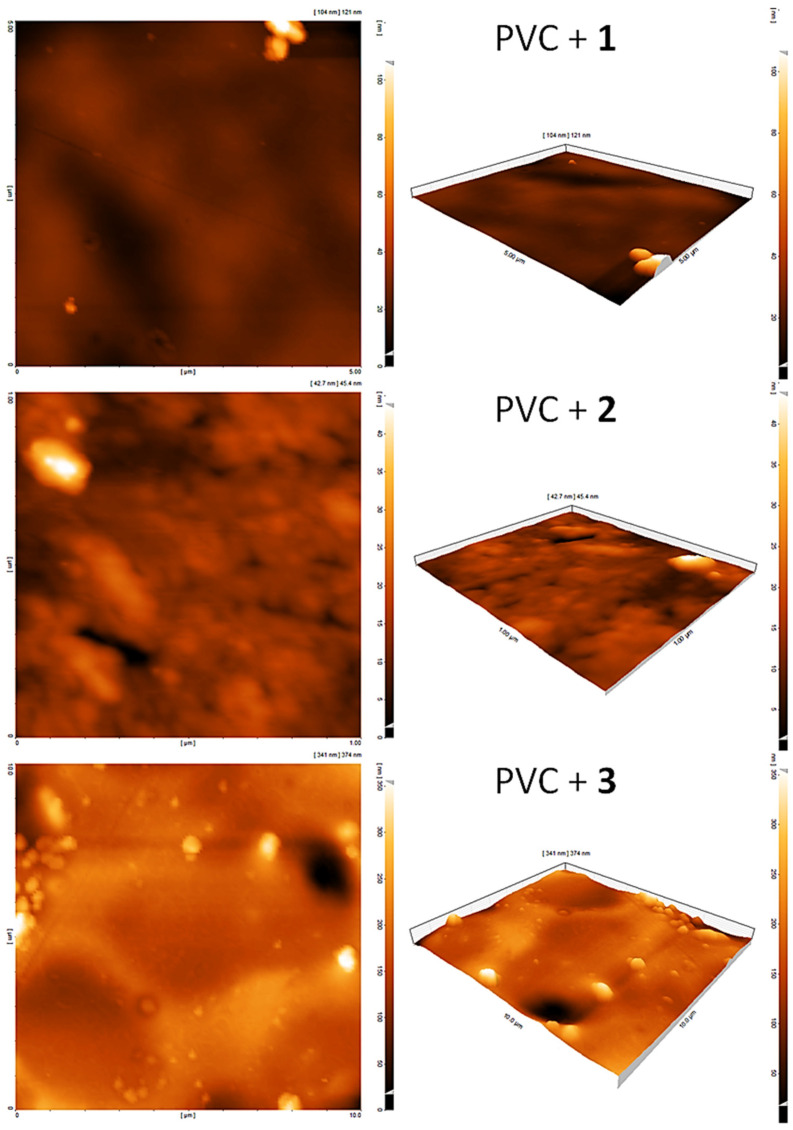
AFM images of PVC films after irradiation.

The roughness of the PVC surface was examined further using AFM, which provides two- and three-dimensional topography. Figure 9 and Figure 10 indicated that the surface of irradiated blends containing **1**–**3** was less rough compared with the pure PVC film. The use of complexes **1**–**3** as PVC additives led to a significant decrease in the rate of both dehydrochlorination and bond breaking of polymeric chains. The roughness factor (*R*q) for irradiated pure PVC was 320.8 compared with 17.4, 37.2, and 41.1 for PVC + **1**, PVC + **2**, and PVC + **3**, respectively. Clearly, the *R*q for the PVC surface decreased significantly in the presence of **1**–**3**. The use of complex **1** led to a drop in the *R*q by 18.4-fold, which is one of the highest compared with those reported [56,57,58,59,60,61,62,63,64]. For example, the fold decrease in the *R*q was 3.3–6.0 using Schiff bases [56,57,58], 5.2–16.6 using organotin complexes [59,60,61,62,63], and 16.8 using polyphosphates [64]. However, the use of tin complexes containing naphthalene sulfonic acid led to a remarkable decrease in the *R*q of PVC up to 59.9-fold, possibly owing to the extended conjugation within the aromatic system of naphthalene [43].

### 3.11. Suggested Mechanisms of the Role Played by Organotin Complexes as PVC Photostabilizers

**Figure 11:**
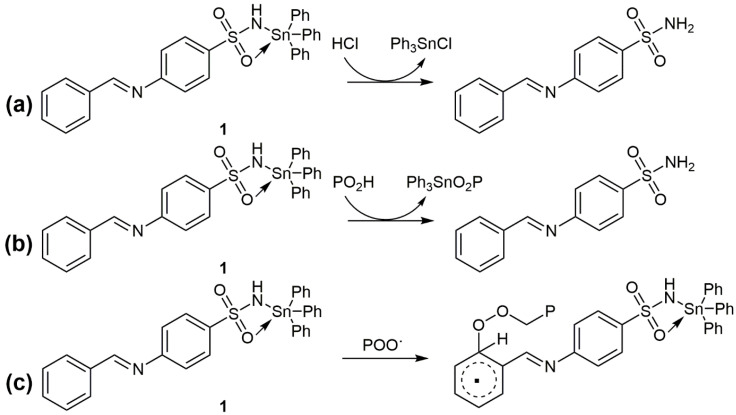
Complex **1** as (**a**) a HCl scavenger, (**b**) a PO_2_H decomposer, and (**c**) a radical scavenger.

The use of tin complexes **1**–**3** as additives leads to a substantial reduction in the degradation and decomposition of PVC films. Complex **1**, which has the highest aromatic content (triphenyl substituents), leads to the most noticeable stabilizing effect, followed by **2** (tri-*n*-butyl substituents) and then **3** (dimethyl substituents). Complexes **1**–**3** act as PVC photostabilizers mainly owing to the presence of aromatic moieties, heteroatoms, and tin atoms. Complexes **1**–**3** can absorb UV light and release the adsorbed energy slowly over time without harming the PVC chains. The highly energetic states produced thanks to the absorption of UV light by PVC can also be stabilized through resonance of aromatic rings [65]. In addition, the tin atom is highly acidic and can scavenge HCl (Figure 11a) [61].

Photooxidation of PVC occurs in the presence of oxygen-containing species such as hydroperoxides (PO_2_H) [66]. Tin complexes inhibit photooxidation by decomposing hydroperoxides (Figure 11b).

Various PVC photooxidation products are generated owing to the formation of peroxide radicals (POO^•^), which are also known as chromophores. Tin complexes **1**–**3** react with chromophores to produce highly stable intermediates (Figure 11c) through resonance of the aromatic rings within additives [66,67]. Therefore, complexes **1**–**3**, and in particular **1**, can inhibit PVC photooxidation and thus enhance the photostability of the polymeric material.

**Figure 12:**
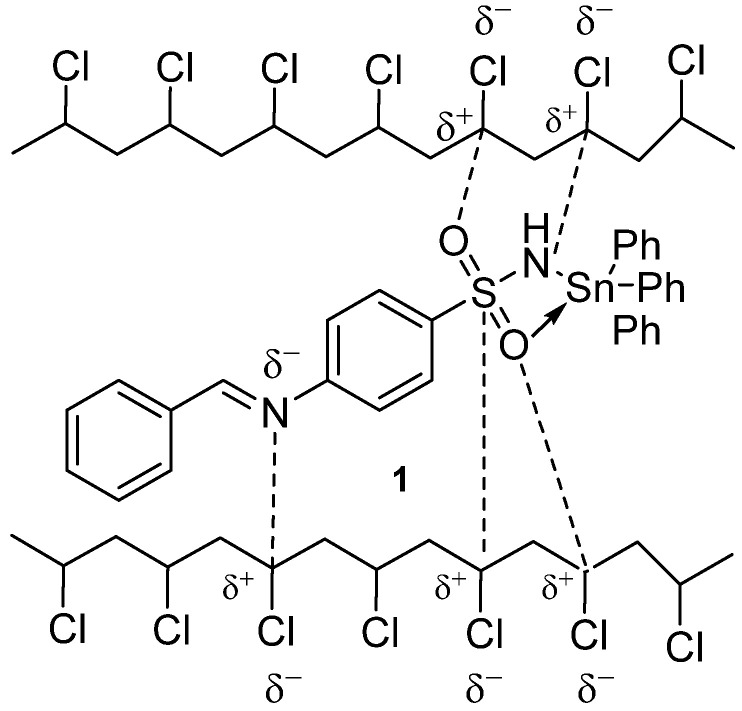
Interaction between complex Ph3SnL and PVC.

The interaction between polarized atoms of the C–Cl bonds in PVC and the N–H, O=S=O, and CH=N bonds in complexes **1**–**3** could stabilize the polymer against photodegradation (Figure 12). Such coordination could help the energy transfer from PVC to tin complexes before it is dissipated. However, such a hypothesis neglects the steric hindrance complications in macromolecules [68].

## 4. Conclusions

Three new tin complexes were synthesized in good yields from the reaction of substituted tin chlorides and 4-(benzylideneamino)benzenesulfonamide. The structures and elemental contents of the tin complexes were established and their surface morphologies were investigated. The polymer weight loss, depression in molecular weight, production of fragments containing a carbonyl group or a C=C double bond, and surface morphology were investigated by comparison of pre- and post-irradiation PVC. The blank PVC film suffers a significant degree of photooxidation and photodegradation as a result of long-term irradiation. On the other hand, PVC blends containing tin complexes showed a reduced level of undesirable changes post-irradiation when compared with the blank film. Thus, the tin complexes synthesized, and in particular the complex containing the triphenyl substituent, act as effective and efficient PVC photostabilizers and enhance photostability.

## Figures and Tables

**Table 1:** Color, melting points, yields, and elemental composition of complexes **1**–**3**.

Complex.	Color	MP (°C)	Yield (%)	Calculated (Found; %)			
				C	H	N	Sn
**1**	Off-White	112–114	86	61.11 (61.16)	4.30 (4.32)	4.60 (4.71)	19.48 (19.55)
**2**	Off-White	132–134	63	54.66 (54.70)	6.97 (7.00)	5.10 (5.12)	21.61 (21.68)
**3**	White	168–170	76	50.39 (50.41)	4.23 (4.16)	8.40 (8.45)	17.79 (17.86)

**Table 2:** Selected FTIR spectral data for some common groups in complexes **1**–**3**.

Complex	Wave Number (*ν*, cm^–1^)						
	NH	CH=N	C=C	SO_2_ asym	SO_2_ sym	Sn–O	Sn–N
**1**	3459	1678	1624	1307	1188	552	440
**2**	3460	1684	1630	1304	1179	533	439
**3**	3461	1667	1623	1307	1185	550	454

**Table 3:** ^1^H-NMR spectral data of **1**–**3**.

Complex	^1^H NMR (500 MHz: δ, ppm, *J* in Hz)
**1**	8.11–8.00 (m, 8H, Ar), 7.55–7.42 (m, 14H, Ar), 7.01 (s, 1H, CH), 6.68 (d, *J* = 7.8 Hz, 2H, Ar), 6.01 (s, exch., 1H, NH)
**2**	7.58–7.52 (m, 5H, Ar), 7.05 (s, 1H, CH), 6.74–6.70 (m, 4H, Ar), 5.94 (s, exch., 1H, NH), 1.79 (t, *J* = 7.4 Hz, 6H, 3CH_2_), 168 (m, 6H, 3CH_2_), 1.46 (m, 6H, 3CH_2_), 1.00 (t, *J* = 7.4 Hz, 9H, 3Me)
**3**	8.08–7.66 (m, 12H, Ar), 7.12 (s, 2H, 2CH), 6.68 (d, *J* = 7.9 Hz, 6H, Ar), 5.95 (s, 2H, exch., 2NH), 1.15 (s, 6H, 2Me).

**Table 4:** ^13^C and ^119^Sn NMR spectral data of **1**–**3**.

Complex	^13^C NMR	^119^Sn NMR
**1**	162.8, 151.9, 143.7, 136.2, 135.7, 130.0, 129.5, 129.2, 129.0, 128.9, 128.7, 121.2, 112.4	−227.4
**2**	161.9, 151.9, 140.3, 135.0, 130.0, 128.6, 127.4, 122.4, 112.4, 27.9, 26.2, 21.0, 13.6	−241.3
**3**	162.8, 151.9, 141.1, 134.6, 132.0, 128.9, 126.9, 121.2, 112.4, 22.8	−143.2

## Data Availability

Data are contained within the article.

## Supplementary Materials

The following are available online at https://www.mdpi.com/article/10.3390/polym13152434/s1, Figure S1: FTIR spectrum of ligand; Figure S2: FTIR spectrum of **1**; Figure S3: FTIR spectrum of **2**; Figure S4: FTIR spectrum of **3**; Figure S5: ^119^Sn NMR spectrum of **1**; Figure S6: ^119^Sn NMR spectrum of **2**; Figure S7: ^119^Sn NMR spectrum of **3**; Figure S8: EDX mapping of PVC before and after irradiation; and Figure S9: EDX mapping of PVC films after irradiation.
