# Supplementary material for: Tin Complexes of 4-(Benzylideneamino)benzenesulfonamide: Synthesis, Structure Elucidation and Their Efficiency as PVC Photostabilizers
Source: Polymers (Basel). 2021 Jul 23;13(15):2434. doi: 10.3390/polym13152434 (PMC8348574; doi:10.3390/polym13152434)
Supplement: Supplementary file 1 [file polymers-13-02434-s001.zip › polymers-1312845-supplementary.pdf]

polymers

MDPI

Tin Complexes of 4-(Benzylideneamino)benzenesulfonamide: Synthesis, Structure Elucidation and Their Efficiency as PVC Photostabilizers

Hassan Ghani [1], Emad Yousif [1], Dina S. Ahmed [2], Benson M. Kariuki [3] and Gamal A. El-Hiti [4,*]

[1] Department of Chemistry, College of Science, Al-Nahrain University, Baghdad 64021, Iraq; st.hassan.ghani@nahrainuniv.edu.iq (H.G.); emad.yousif@nahrainuniv.edu.iq (E.Y.)
[2] Department of Medical Instrumentation Engineering, Al-Mansour University College, Baghdad 64021, Iraq; dina.saadi@muc.edu.iq
[3] School of Chemistry, Cardiff University, Main Building, Park Place, Cardiff CF10 3AT, UK; kariukib@cardiff.ac.uk
[4] Department of Optometry, College of Applied Medical Sciences, King Saud University, P.O. Box 10219, Riyadh 11433, Saudi Arabia
* Correspondence: gelhiti@ksu.edu.sa; Tel.: +966-11469-3778; Fax: +966-11469-3536

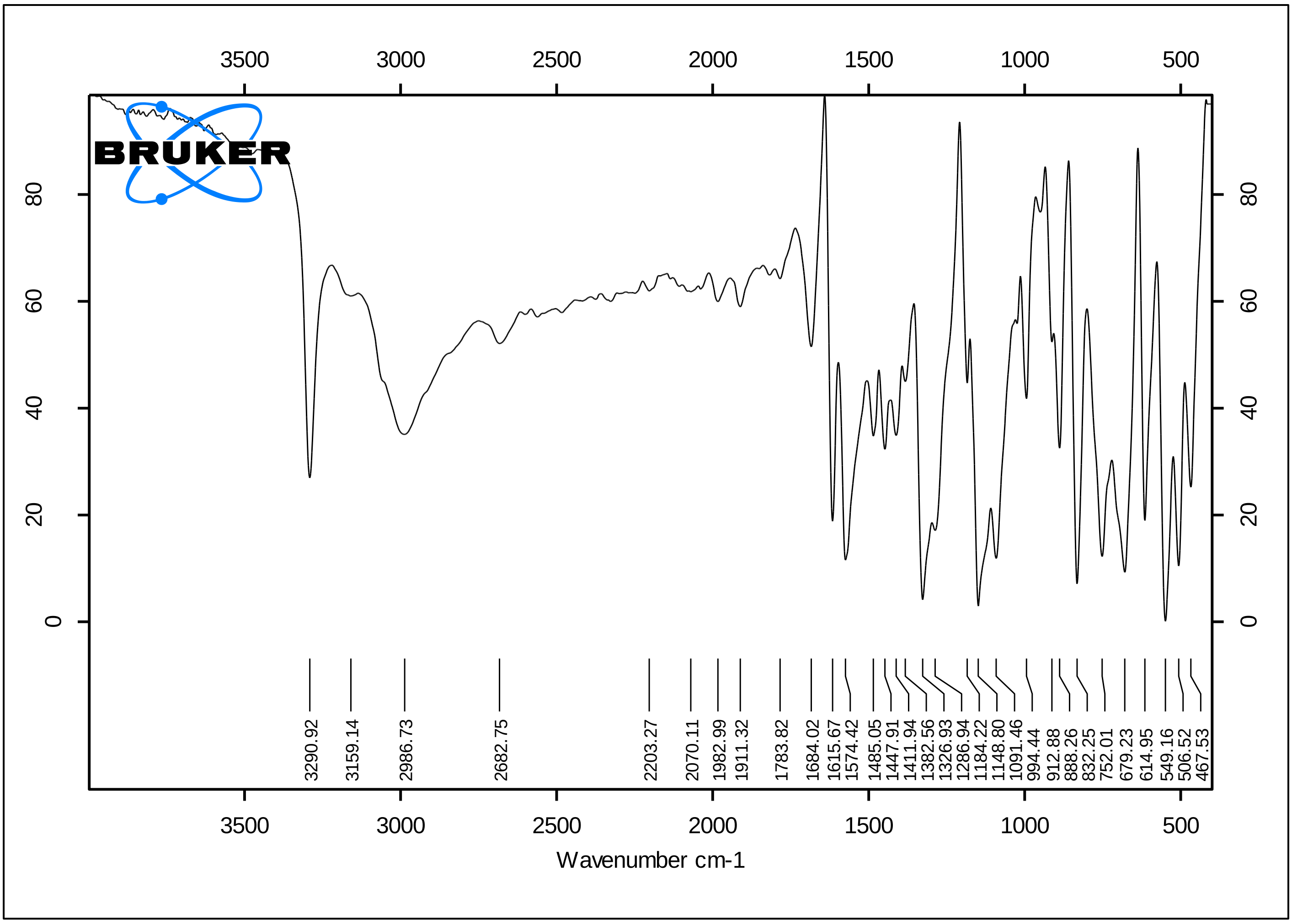

Figure S1. FTIR spectrum of 4-(benzylideneamino)benzenesulfonamide.

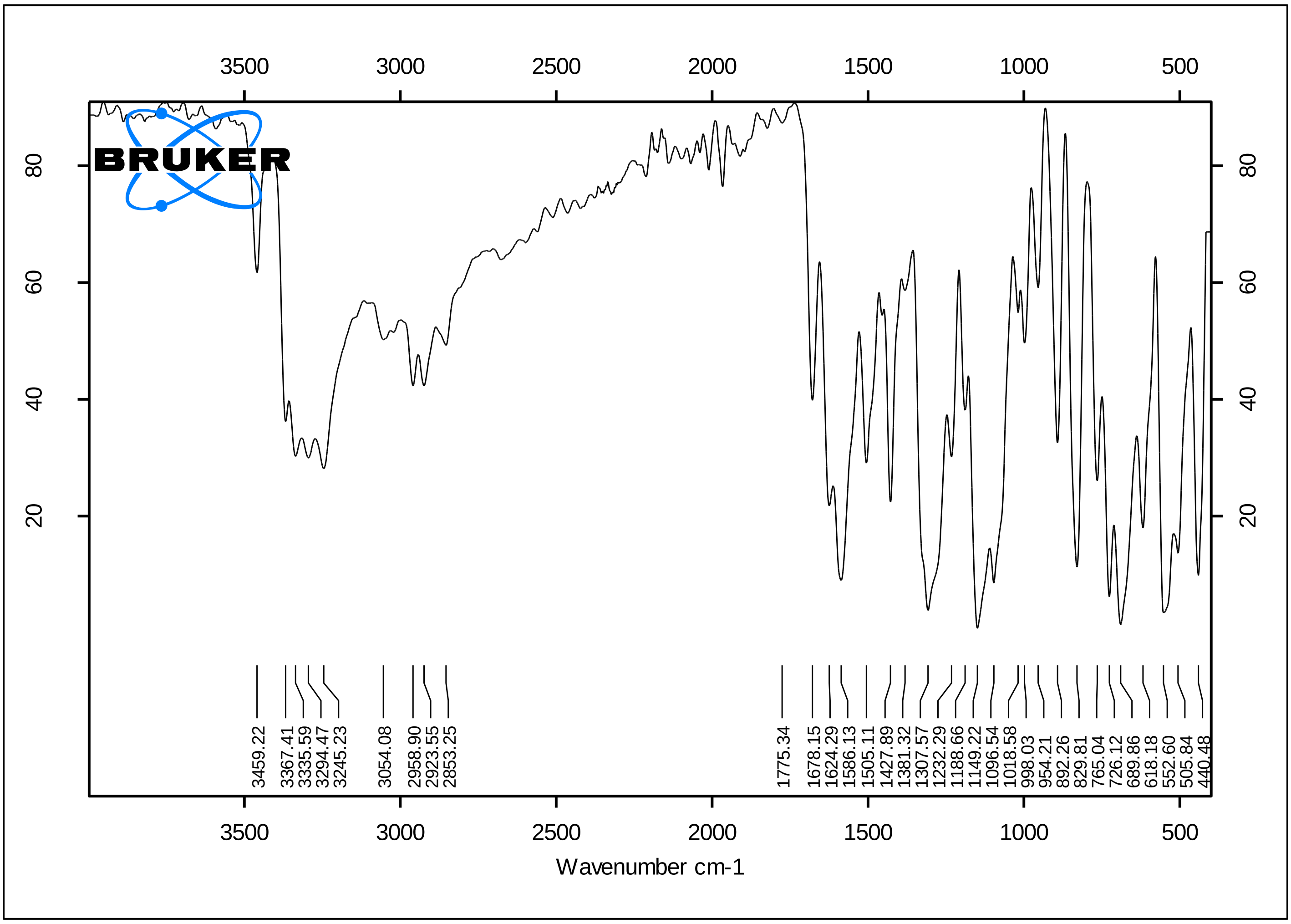

Figure S2. FTIR spectrum of organotin complex **1**.

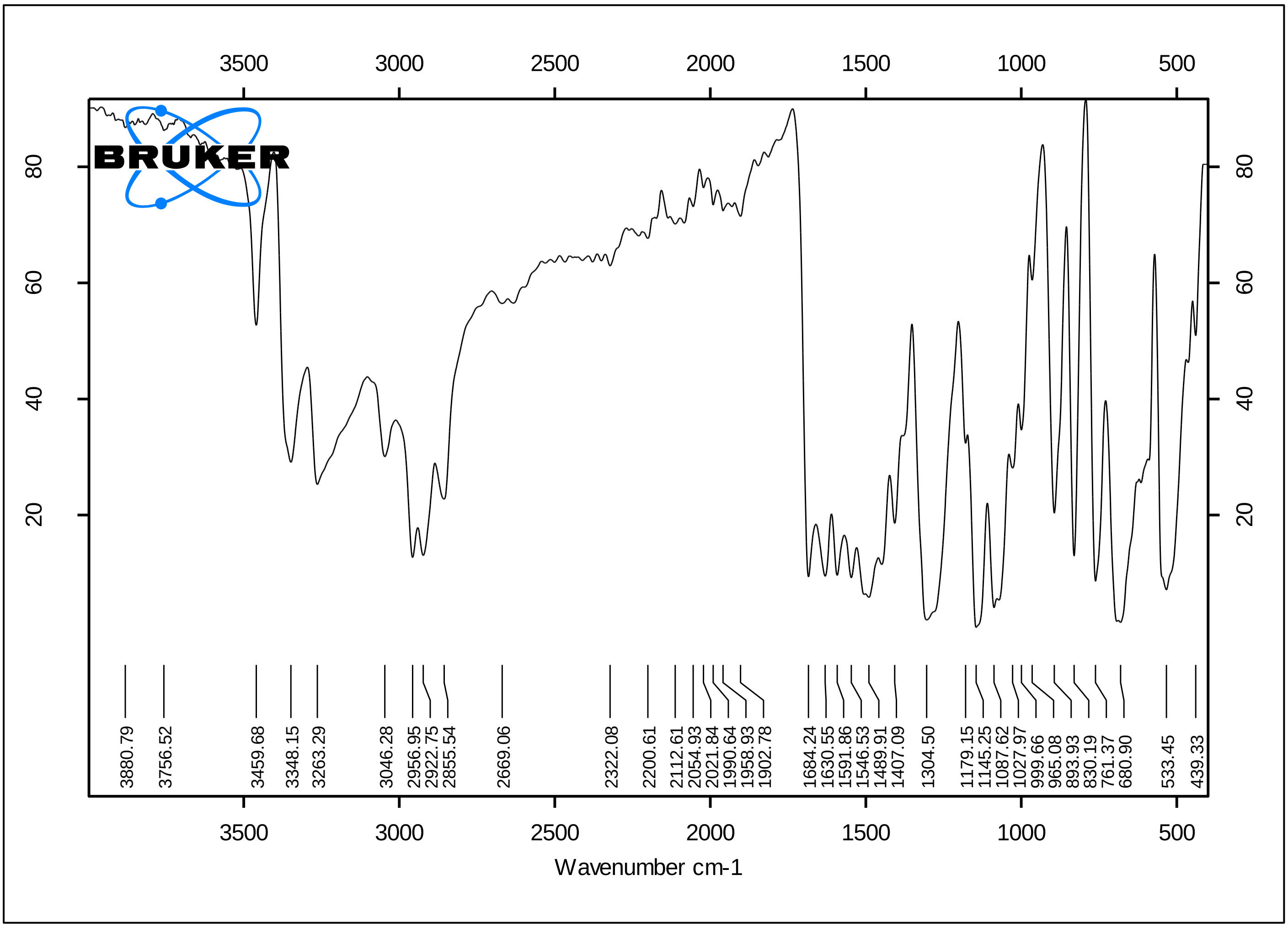

Figure S3. FTIR spectrum of organotin complex **2**.

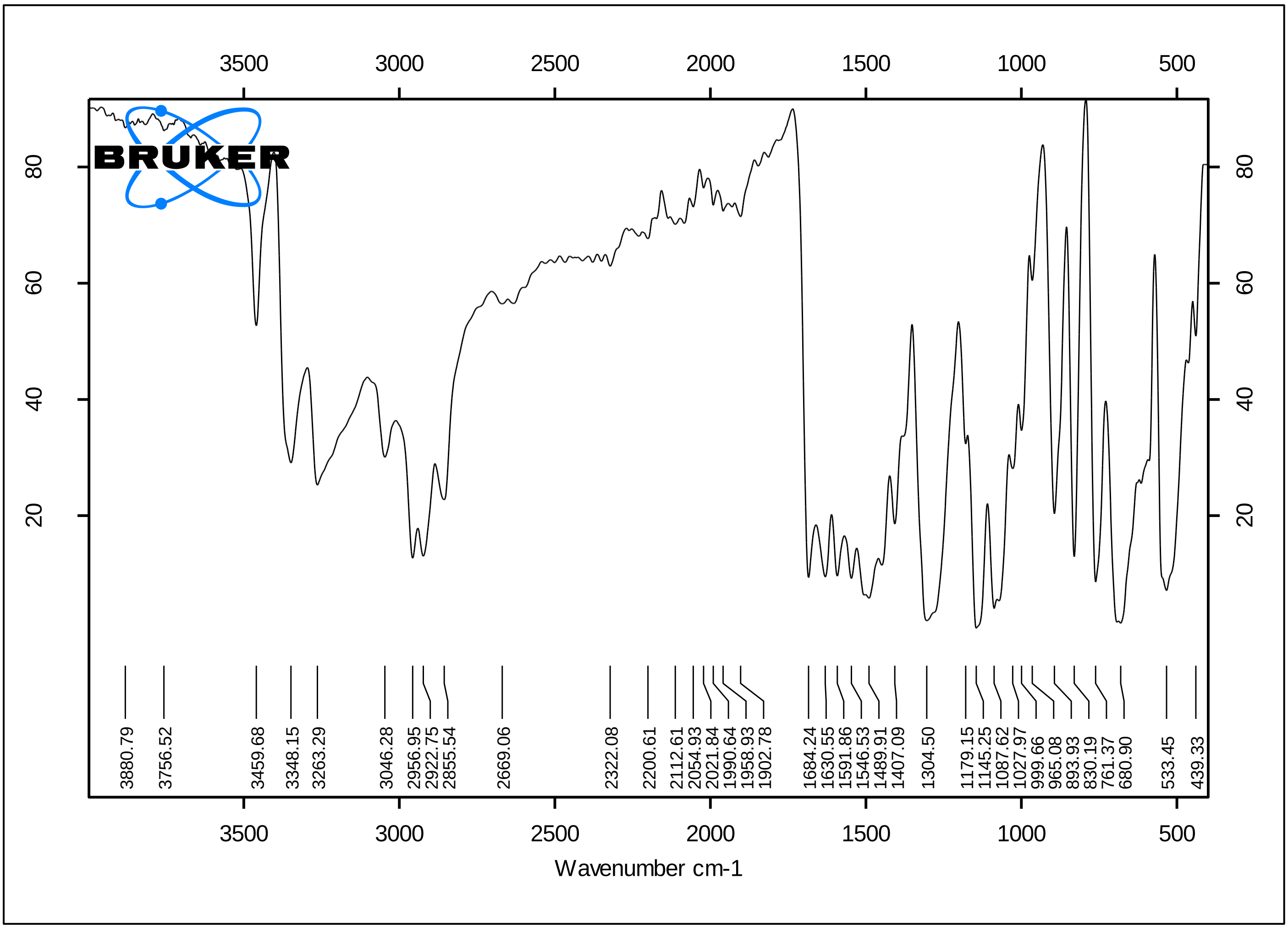

Figure S4. FTIR spectrum of organotin complex **3**.

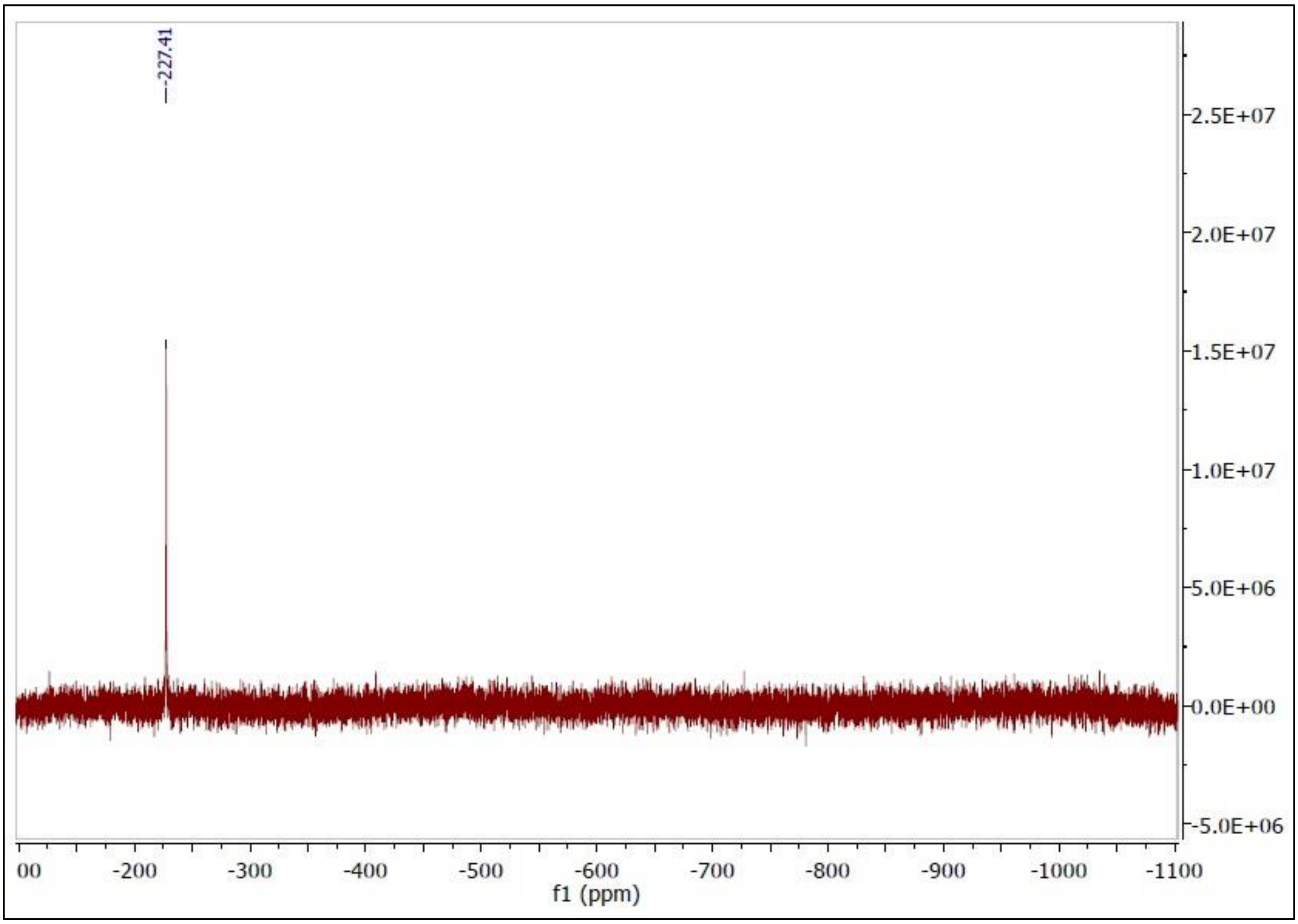

Figure S5. ^{119}Sn spectrum of organotin complex **1**.

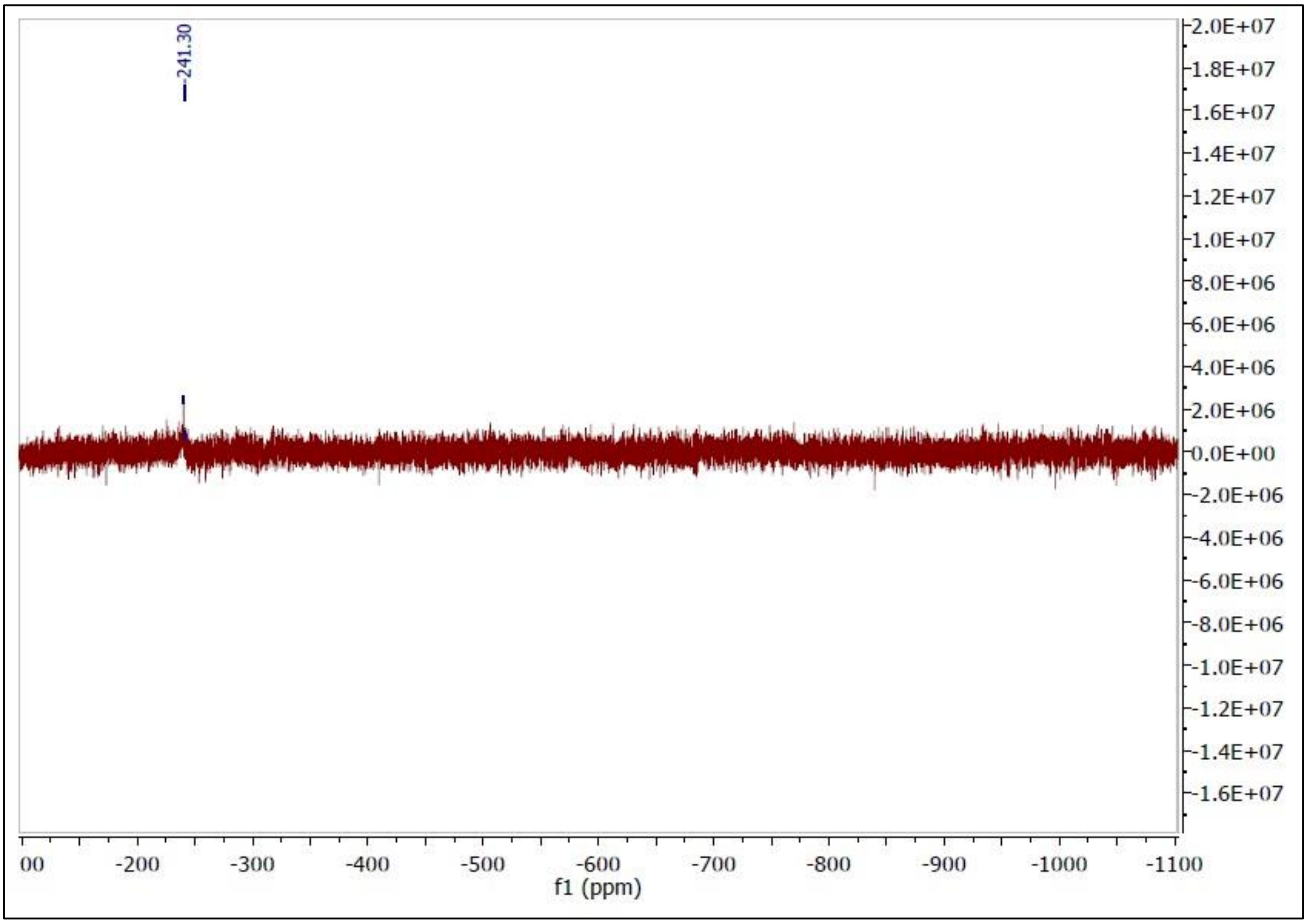

Figure S6. ^{119}Sn spectrum of organotin complex **2**.

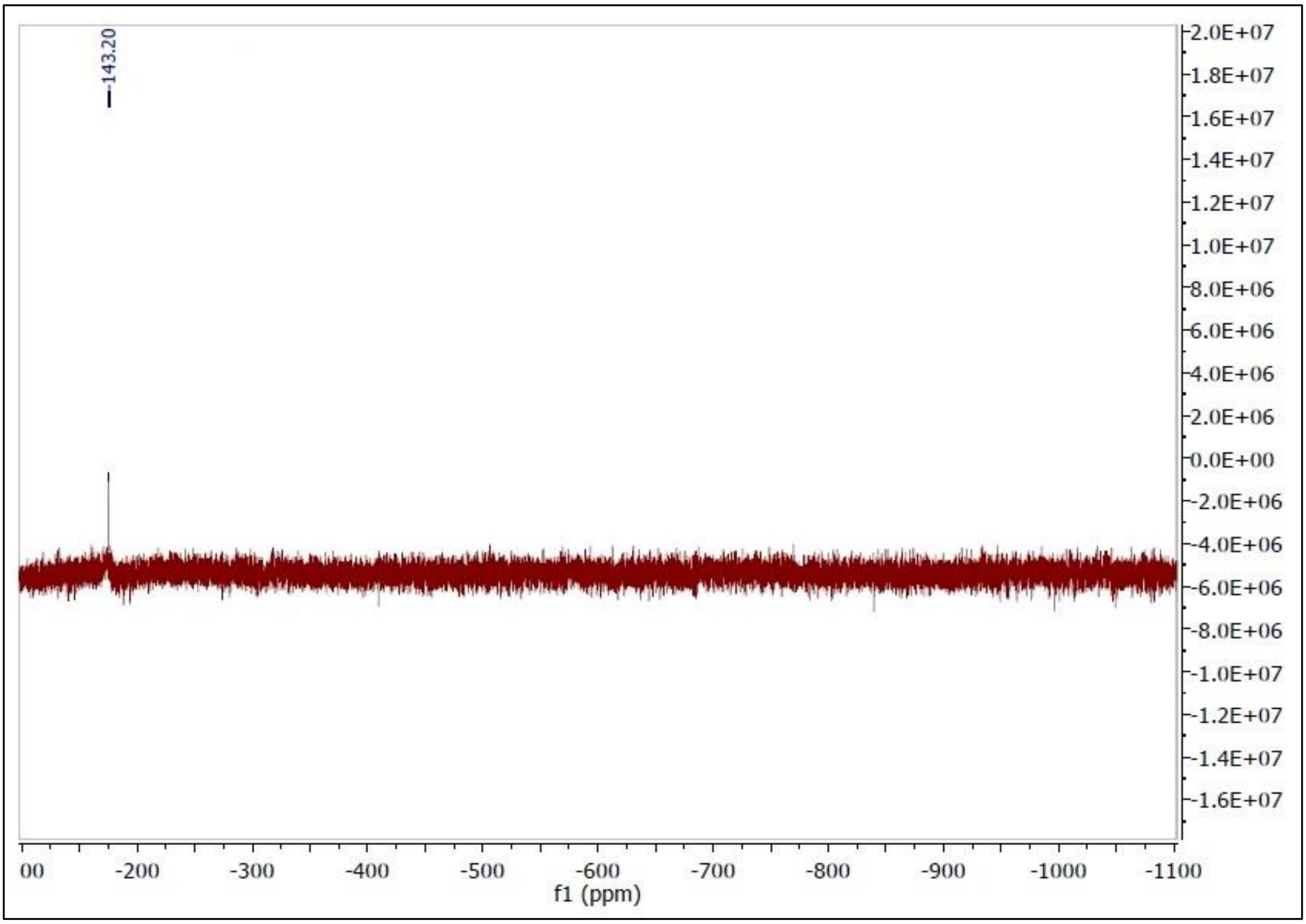

Figure S7. ^{119}Sn spectrum of organotin complex **3**.

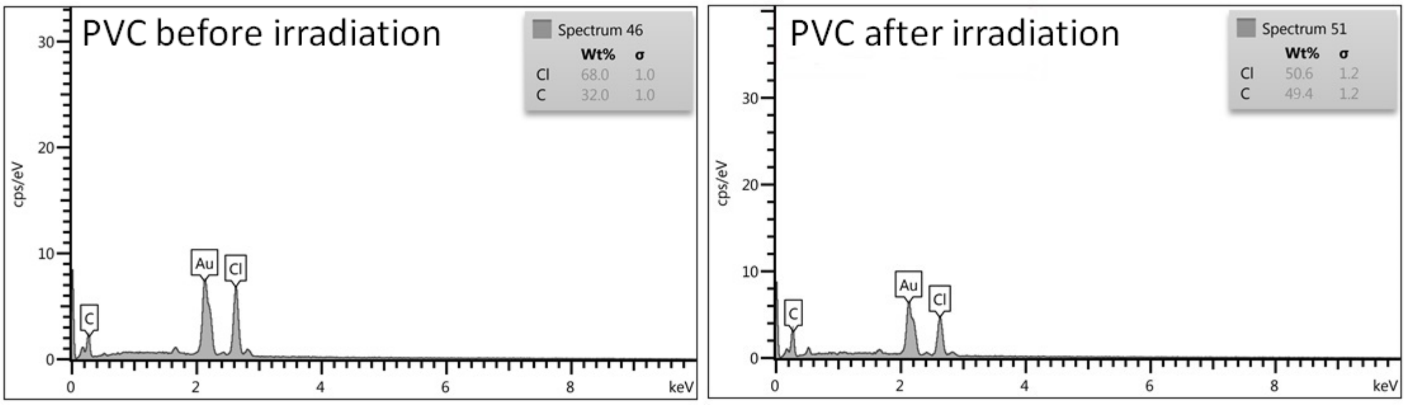

Figure S8. EDX mapping of PVC before and after irradiation.

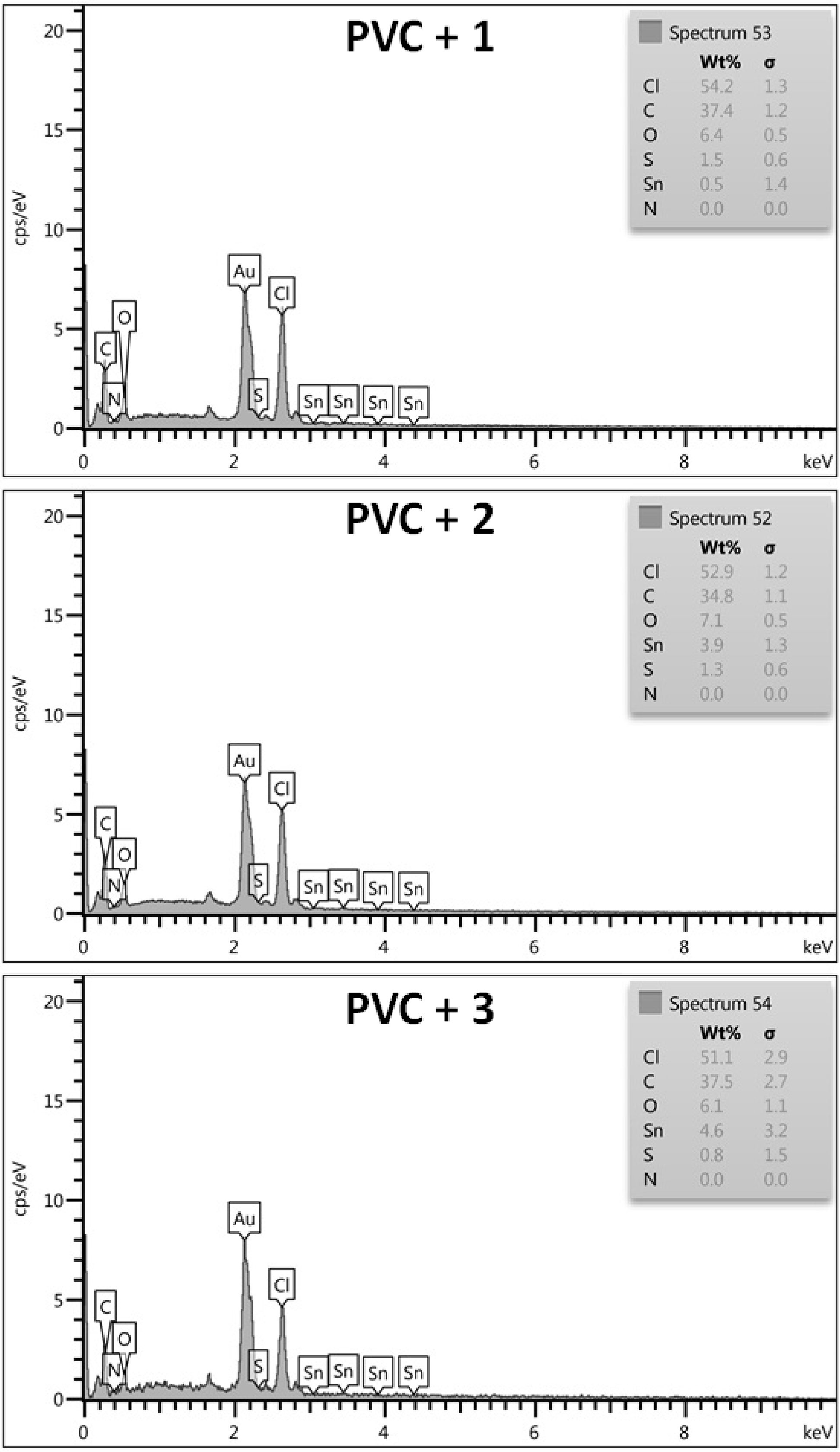

Figure S9. EDX mapping of PVC films after irradiation.
